# Evaluation of the Composition of the Cholesterol, Tocopherols, β-Carotene and Fatty Acids Profile of the Liver Tissue of Male Water Buffaloes (*Bubalus bubalis*) Managed in Different Ecosystems of the Eastern Amazon

**DOI:** 10.3390/ani13243785

**Published:** 2023-12-08

**Authors:** Laurena Silva Rodrigues, Jamile Andrea Rodrigues da Silva, José de Brito Lourenço-Júnior, André Guimarães Maciel e Silva, Thomaz Cyro Guimarães de Carvalho Rodrigues, Welligton Conceição da Silva, Thiago Carvalho da Silva, Vinicius Costa Gomes de Castro, Cristina Mateus Alfaia, André Martinho de Almeida, José António Mestre Prates

**Affiliations:** 1 Postgraduate Program in Animal Science (PPGCAN), Institute of Veterinary Medicine, Federal University of Para (UFPA), Castanhal 68746-360, Brazil; laurenazootec@gmail.com (L.S.R.); joselourencojr@yahoo.com.br (J.d.B.L.-J.); andregms@gmail.com (A.G.M.e.S.); thomazguimaraes@yahoo.com.br (T.C.G.d.C.R.); 2 Institute of Animal Health and Production, Federal Rural University of the Amazônia (UFRA), Belem 66077-830, Brazil; jamileandrea@yahoo.com.br (J.A.R.d.S.); timao22@hotmail.com (T.C.d.S.); 3 Postgraduate Program in Animal Health and Production in the Amazon (PPGSPAA), Federal Rural University of the Amazon (UFRA), Belem 66077-830, Brazil; vinicius.c.gomes@hotmail.com; 4 Center for Interdisciplinary Research in Animal Health (CIISA), Faculty of Veterinary Medicine, University of Lisbon, 1300-477 Lisboa, Portugal; cpmateus@fmv.ulisboa.pt (C.M.A.); japrates@fmv.ulisboa.pt (J.A.M.P.); 5 LEAF—Linking Landscape, Environment, Agriculture and Food Research Center, Associate Laboratory TERRA, Instituto Superior de Agronomia, Universidade de Lisboa, Tapada da Ajuda, 1349-017 Lisboa, Portugal; aalmeida@isa.ulisboa.pt; 6 Associate Laboratory for Animal and Veterinary Science (AL4Animals), 1300-477 Lisboa, Portugal

**Keywords:** Amazon, tissue composition, nutritional quality, ruminants, liver, extensive and intensive systems

## Abstract

**Simple Summary:**

This study evaluated the influence of Amazonian ecosystems, during the dry and rainy seasons, on the composition of the livers of water buffaloes (*Bubalus bubalis*) raised in the Eastern Amazon in extensive and intensive systems. The results showed that the total lipid content was affected by location and season. Total cholesterol, α-tocopherol and γ-tocopherol were influenced by the interaction between ecosystems, location and seasonal periods. Animals raised in pastures had higher levels of omega-3, while those in confinement had higher levels of omega-6. Amazonian ecosystems influenced the nutritional values of buffalo livers, with better results in the extensive system.

**Abstract:**

The diet offered to animals has a great influence on the composition of tissues and, consequently, the quality. The objective of this study was to evaluate the influence of Amazonian ecosystems, in the dry and rainy periods of the year, on the composition of cholesterol, tocopherols, β-carotene and the fatty acid profile of the livers of water buffaloes (*Bubalus bubalis*) reared in the Eastern Amazon, in an extensive or intensive system. Total lipid content was influenced by the location and time of year (*p* < 0.05). Ninety-six male water buffaloes were used (12 per sampling period), aged between 24 and 36 months, with average weights of 432 kg (end of the rainy season) and 409 kg (end of the dry season). Total cholesterol, α-tocopherol and γ-tocopherol influenced the relationship between extensive vs intensive ecosystems, location, periods and the interaction between the location and period of the year (*p* < 0.05). Animals raised in a pasture ecosystem had the highest values of omega-3, and those raised in confinement, the highest values of omega-6 (*p* < 0.05). The proportions of n-6/n-3 and hypocholesterolemia (7.14) and hypercholesterolemia (3.08%) (h/H) were found in greater amounts in animals raised in confinement (*p* < 0.05). The atherogenic index (AI) had a higher value in the rainy season, in animals raised in Santarém (2.37%), with no difference between pasture and feedlot ecosystems, except in animals raised in the rainy season in Nova Timboteua, with a lower AI (1.53%). The thrombogenicity index (TI) was higher in the livers of confined animals (0.32%) and lower (0.18%) in those raised in Nova Timboteua (rainy season). Amazonian ecosystems influence the nutritional values of buffalo liver, with the best nutritional values in animals in the extensive system.

## 1. Introduction

The Amazon, the largest biome in the world, occupies 49% of the territory of Brazil, and, due to its great area, it is not an homogeneous biome, as it is, in fact, a set of distinct ecosystems that interact and form a larger unit [1,2]. These ecosystems that comprise it differ in characteristics such as: altitude, temperature, humidity, rainfall concentration, droughts and river floods, flooded areas, incidence of sunlight and soil depth [3,4].

These variations influence the composition of the soil and pastures throughout the year, which, in turn, influence the production strategies and the composition of the animals’ tissues [5,6,7]. This occurs because the main ruminant rearing system in these areas is extensive: in native pasture ecosystems in areas subject to flooding, distributed on the island of Marajó; native to flooded areas, in the Lower and Middle Amazon microregion; and cultivated on unflooded areas, in areas originally forested [6,7,8,9,10].

Characteristic of this region, buffalo farming has been one of the sources of food and income for the local population since their arrival in 1890 [11]. The Brazilian buffalo herd is 1.6 million strong and more than 70% are raised in the Amazon [12]. The animals have good performance in this region, considering their lower susceptibility to diseases commonly observed in other species [13,14], excellent feed conversion and high potential for meat and milk production, in addition to their use in agriculture for animal traction; there are even studies that point to their resistance to thermal stress [15,16,17].

The better feed conversion rate of buffaloes is a notable feature that sets them apart from other livestock animals. This is largely due to their anatomy and specialized digestive system. Buffaloes have the ability to efficiently extract nutrients from even low-quality forages, which means they can feed on pastures that may not be suitable for other ruminants such as cattle. This ability to make the most of more abundant and accessible food resources contributes to the sustainability of buffalo meat and milk production, especially in regions where high-quality pastures may be limited [16].

Furthermore, buffaloes also excel in terms of their excellent thermoregulatory response. They are well adapted to hot and humid environments, such as many areas in the Amazon region. Buffaloes have more efficient sweat glands than cattle, allowing them to dissipate heat more effectively. Additionally, their ability to enter water and remain partially submerged helps cool their bodies. These physiological adaptations make buffaloes more resilient to thermal stress, a significant advantage in tropical climates where high temperatures can negatively impact the performance of other livestock species [17,18,19].

However, research that characterizes the influence of ecosystems on the soil, vegetation and the composition of the edible parts of this species, such as the liver, is scarce. The use of this organ in human nutrition involves the ingestion of high-quality proteins, fat, and a high content of minerals, vitamins and fatty acids, being the edible organ that is richest in nutrients, along with the muscles [20]. Therefore, information on the composition of cholesterol, tocopherols, β-carotene and the fatty acid profile of the buffalo liver and the variation in composition depending on diet is limited.

The objective of this study was to quantify the levels of total cholesterol, tocopherols, β-carotene and the fatty acid profile of the buffalo liver in ecosystems of native pastures, cultivated areas and buffaloes in confinement in the Eastern Amazon in the dry and rainy seasons of the year. Our hypothesis is that ecosystems and seasonal change in the Eastern Amazon may influence the levels of total cholesterol, tocopherols, β-carotene and the fatty acid profile at the liver level in water buffalo.

## 2. Materials and Methods

### 2.1. Ethical Aspects

This research was approved by the Ethics Committee for the Use of Animals (CEUA), with protocol number 4542190820, granted by the Federal Rural University of Amazônia (UFRA).

### 2.2. Experimental Animals

A prospective study was carried out. The work used samples of liver tissue from crossbred water buffaloes (Murrah/Mediterranean), that were clinically healthy, from three breeding ecosystems in the Eastern Amazon (extensive production system), and from animals raised in an intensive production system (confinement), in two seasons of the year (rainier and less rainy periods). Only male water buffaloes were used, as they are most commonly slaughtered in the study region due to their ability to reach a higher weight quickly compared to females. Ninety-six male water buffaloes were used (12 per sampling period), aged between 24 and 36 months, with average weights of 432 kg (end of the rainy season) and 409 kg (end of the dry season). The sample was the same as that used in the study by Rodrigues et al. [6], Rodrigues et al. [7] and Silva et al. [8]. Tissue samples were collected in commercial slaughterhouses, in each of the considered ecosystems. Table 1 and Table 2 show the composition of diets and soil characteristics, respectively.

### 2.3. Ecosystems Studied

The Amazon region is made up of about 5 million km^2^ of which the state of Pará is about 1.24 million km^2^ [12]. This large territorial region allows for a differentiated climate and vegetation, as described by Biel et al. [21], as well as different systems and strategies for raising animals. Table 1 and Table 2 demonstrate this diversity, with variations in soil and vegetation composition between ecosystems and within the same ecosystem, with the change in season. The experimental treatments involved three Amazon buffalo breeding ecosystems, and an intensive system (confined).

### 2.4. Rainfall and Diets

Ecosystem 1(Lower Amazon) —Rainier period between January and June, annual average rainfall of 2500 mm. Feeding exclusively on pasture, basically composed of *Panicum elephantpes*, *Leersia hexandra* and *Hymenachne amplexicaulis* (grasses native to floodplains).

Ecosystem 2 (Marajo)—Wettest period between December and May, annual average rainfall of 2000 mm. Buffalo also in a traditional (extensive) rearing system, fed on native pasture and some cultivated grasses (*Bhachiaria Brizantha* and *Panicum maximum* cv Mombaça).

Ecosystem 3 (Nova Timboteua) —Wettest period between December and August, annual average rainfall of 2467 mm. Diet based on upland cultivated pastures (*Bhachiaria humidicola* and *Panicum maximum* cv. Mombaça).

Confinement (Intensive)—Wettest period between December and May, annual average rainfall of 2030 mm. Animals fed roughage (sorghum silage) and concentrate (soybean meal, wet sorghum premix and commercial feed).

### 2.5. Samples and Chemical Analysis of Diets

In the pasture, samples of 1 m^2^ were collected at five different points, which were homogenized, and then about 1 kg of sample was taken from each ecosystem and sent to the laboratory. In addition, samples of the ingredients and diet of the feedlot animals were collected. The analyses were carried out at the Laboratory of Animal Nutrition, the Federal University of Pará/Campus Castanhal, Pará, Brazil.

These samples were dried in a forced ventilation oven (55 to 60 °C) for 72 h to avoid the loss of volatile compounds and chemical changes, then cooled to room temperature and ground in a Willey mill at 1 mm for analysis: dry matter (INCT-CA G-003/1 method); ash (INCT-CA M-001/1); total nitrogen (INCT-CA N-001/1 method—quantified using a three-step micro Kjeldhal method; digestion with sulfuric acid, basic distillation and titration with hydrochloric acid, and the value obtained multiplied by 6.25 to obtain the crude protein); neutral detergent fiber (INCT-CA F-001/1 and INCT-CA F-002/1) and acid detergent fiber (INCT-AC F-003/1 and INCT-AC F-004/1), both corrected for protein and ash, according to the methods recommended by the National Institute of Science and Technology in Animal Science (INCT-CA) [22].

The determination of non-fiber carbohydrates was according to [23] and the TDN by using the Clemson University equation: TDN = 93.59 − (FDA × 0.936).

### 2.6. Liver Tissue Collection and Preparation

Liver tissue samples were collected from all buffalo belonging to each experimental group (ecosystems and periods of the year). Collection took place right after slaughter, removing approximately 100 g (wet weight) of tissue and storing it in a freezer at −80 °C, until lyophilization. Subsequently, the samples were lyophilized to constant weight (about 48 h) with Christ Alpha 1–2 LDplus lyophilizer (Christ alpha, OsterodeamHarz, Germany).

### 2.7. P Determination of Cholesterol, β-Carotene and Vitamin E

Simultaneous analysis of meat total cholesterol, β-carotene and vitamin E homologues was performed according to the methodology defined in detail in [24]. Analysis was performed by high-performance liquid chromatography (HPLC) using a normal phase silica column (Zorbax Rx-Sil column, 5 µm particle diameter, 4.6 mm ID × 25 cm, Agilent Technologies Inc., Santa Clara, CA, USA) and two serial detectors (diode array and fluorescence). Quantification was based on the external standard method using the peak area versus concentration calibration curves.

### 2.8. Determination by HPLC in Normal Phase

The temperature used to determine the HPLC was 20 °C. The mobile phase was hexane–isopropanol (99:1) (flow: 1.0 mL/min) with an injection volume of 20 µL for α-tocopherol and 100 µL for the remaining tocopherols, and 20 or 100 µL for cholesterol. The detection was to tocopherols (e.g., 295 nm, em. 325 nm, PMT-Gain 14), β-carotene (450 nm), all-trans-retinol (325 nm) and cholesterol (202 nm).

### 2.9. Calculations and Control of Methods

Cholesterol, β-carotene and tocopherols were quantified by the external standard method. For the tocopherol profile (α-tocopherol, β-tocopherol, γ-tocopherol and ∆-tocopherol), four calibration curves were used, one for cholesterol and another for β-carotene, respectively, of areas versus concentration.

### 2.10. Lipid Extraction and Fatty Acid Methylation and Analysis

Total lipids were extracted in duplicate [25], with modifications of Carlson [26], and determined gravimetrically by weighing the lipid residue after solvent evaporation. Fatty acid methyl esters (FAME) were obtained, as previously described in [27]. Then, FAME were extracted with n-hexane and analyzed by gas chromatography equipped with a flame ionization detector (GC-FID) (HP 7890A chromatography; Hewlett-Packard, Avondale, PA, USA) and separated using a fused capillary column (Supelcowax^®^ 10, 30 m, 0.2 mm id, film thickness of 0.20 mm; Supelco, Bellefonte, PA, USA). The chromatography conditions were previously reported in [28]. Nonadecanoic acid methyl ester (19:0; Sigma, San Luis, EUA) was added as an internal standard. Fatty acid identification was obtained by comparison with a commercial standard FAME mixture of 37 components (Supelco, Inc., Bellefonte, PA, USA). Fatty acids were expressed as g/100 g of total fatty acids.

### 2.11. Nutritional and Health Lipid Indices

To determine the nutritional and healthy lipid indices of meat, the following equations were used (in the fatty acid profile) [29,30,31] (Table 3).

### 2.12. Statistical Analysis

The experimental design was a completely randomized 3 × 2 + 1 factorial arrangement (three production systems, two seasonal periods and an additional treatment corresponding to the confinement in feedlot). The variables were analyzed in the PROC MIXED of SAS version 9.1 (2014) (SAS Institute Inc., Cary, NC, USA), considering the following model: yijk = µ + αi + βj + γij + ԑijk and yh = µ + δα + Ԑh, where:

yijk: variable response related to the level of the first factor (i = 1, 2 and 3) combined with the level of the second factor (j = 1, 2) in a specific repetition (k = 1, 2, … r);

µ: general mean;

αi: effect of the first factor level (local—production systems) (i);

βj: effect of the second factor level (seasonal periods) (j);

γij: effect of the interaction of the first factor level (i) with the second factor level (j);

ԑijk: experimental error associated with observation yijk and assumption that ԑĳk~N (0, σ2) with an independent structure;

yh: variable response associated with the observation (h = 1.2, …, m) of additional treatment (confinement in feedlot).

δα: effect of the additional treatment.

Ԑh: experimental error associated with additional treatment with the assumption that Ԑh~N (0, σ2) with an independent structure.

The production systems (extensive and intensive) and the seasonal period (dry and rainy season) were considered fixed effects. Extensive and intensive production systems, as well as the interaction between location and time of year, were considered contrasts. The Tukey–Kramer post hoc test was applied, as the data were unbalanced due to losses. The significance level α = 0.05 was adopted to assess the differences between the least squares means of each group.

The statistics used aimed to preserve the differences between the existing extensive production systems. This is a relevant aspect, as the production and field conditions of extensive systems are considerably different from each other. In addition, they are in regions with significant differences in several aspects, and in an extremely large geographic area (Brazilian Amazon). Indeed, each (extensive) breeding system represents a different reality.

## 3. Results

### 3.1. Lipids, Cholesterol and Vitamins

There was no difference in the total lipid content (mg/g of liver) between rearing systems (extensive vs intensive) (*p* = 0.06) and location vs period (*p* = 0.06) (Table 4). However, there was a difference in location (*p* < 0.01), with the highest mean in the Lower Amazon (41.08), followed by Nova Timboteua (34.08), Marajó (33.71) and confinement (32.61). The period also influenced the lipid content in Marajó (*p* = 0.02), with an increase of 2.74 in the rainy season, and Nova Timboteua, with an increase of 5.74 (mg/g of liver) in the rainy season.

The livers of water buffaloes raised in the dry season in the Lower Amazon and in the rainy season in Nova Timboteua had the highest cholesterol levels, 2.57 and 2.49, respectively, but did not differ from animals raised in the rainy season in the Lower Amazon and Marajó (*p* < 0.05). The lowest cholesterol value was found in buffalo raised in the dry season of Marajó (*p* < 0.01).

With regard to α-tocopherol contents, the highest value was found in the rainy season in the Lower Amazon (33.91%) and the lowest in water buffaloes raised in the dry season in Marajó (8.50%) (*p* < 0.05). The livers of animals raised in the rainy season in the Lower Amazon showed a higher value of γ-tocopherol (1.17), not differing from the animals raised in Nova Timboteua during the rainy season (*p* < 0.05).

The highest levels of γ-tocotrienol were observed in the livers of buffalo raised in confinement (1.49%), which did not differ from those found in the pasture ecosystems of Marajó and Nova Timboteua in the two periods of the year.

### 3.2. Fatty Acids

Lauric acid (C12:0) was higher in animals raised in Nova Timboteua (0.16—dry period), and did not differ from animals raised in the dry period in the Lower Amazon (*p* < 0.05) (Table 5). The livers of water buffaloes from the Lower Amazon (PC) showed a higher value of myristic acid (C14:0) (0.47) (*p* < 0.05), which did not differ from that of animals from the Lower Amazon (PS) and in both periods, in Nova Timboteua and those in confinement. As for myristoleic acid (C14:1 c9), the highest content was found in the livers of confined animals (0.06), and did not differ from the values determined in water buffaloes from the Lower Amazon in the dry period of the year, or in Marajó in the rainy period of the year (*p* < 0.05).

Pentadecylic acid (C15:0) was higher in DS from Nova Timboteua (0.61), and palmitic acid (C16:0) in animals raised in the Lower Amazon (RS—19.52), not differing from pasture ecosystems, except in animals reared in Nova Timboteua (RS—15.49) and feedlot (15.58), which had the lowest value (*p* = 0.03). The tissue with the highest amount of palmitoleic acid (C16:1 c9) was that of animals raised in the Lower Amazon (RS—1.05), and in the rainy season in Nova Timboteua (1.06). The levels of margaric saturated acids (C17:0) were higher in the livers of buffalo raised in the Lower Amazon (DS—3.78%) and the lowest value was observed in feedlot animals (0.88) (*p* < 0.05).

Feedlot animals had the lowest C17:1 c9 fatty acid content (0.28) and the highest stearic acid value (C18:0) (25.60); however, C18:0 did not differ from the extensive system. C18:1 monounsaturated acid was higher in Nova Timboteua water buffaloes, in both periods of the year, and lower in confinement (2.91) (*p* < 0.01). The lowest value of oleic acid (C18:1 c9) occurred in animals from Nova Timboteua (DS—14.69), and the highest levels of linoleic acid (C18:2 n-6) and gamma-linolenic acid (C18:3 n-6) were found in the livers of water buffaloes raised in feedlot (12.06 and 0.37, respectively) (*p* < 0.05). α-linolenic acid (C18:3 n-3) was highest in animals from Nova Timboteua (RS, 1.98), and lowest in confined animals (0.47).

The highest percentage (1.63) of conjugated linoleic acid, CLA (C18:2 c9,t11), was observed in the livers of animals from the Lower Amazon (PC), and the lowest value was found in feedlot animals (0.57) (*p* < 0.01). Arachidic saturated acid (C20:0) was higher in water buffaloes raised in Marajó (RS—0.15%), and did not differ from animals in the Lower Amazon in both periods of the year. Eicosanoic acid (C20:1 c11) was lower in animals reared in Marajó (PS) (*p* < 0.05).

In buffaloes in confinement, the highest levels of dihomo-gamma-linoleic acid (C20:3 n-6, ADGL) and arachidonic acid (C20:4 n-6, AA) were observed, with 4.40 and 8.55, respectively, and the lowest levels of eicosapentaenoic acids (C20:5 n-3, AEP) and docosapentaenoic acid (C22:5 n-3), 0.81 and 2.07, respectively (*p* < 0.05). Docosahexaenoic acid (C22:6 n-3, ADH) was higher in Marajó (PS) and lower in feedlot animals (*p* < 0.01). DMA contents (18:0 and 18:1) were higher in feedlot animals, with 0.11 and 0.03, respectively (*p* < 0.05).

### 3.3. Partial Sums of Fatty Acids

The sum of saturated fatty acids (ΣSFA) was highest in the livers of Lower Amazon water buffaloes (47.67%) and lowest in feedlot (42.75%) (*p* < 0.05) (Table 6). The sum of monounsaturated fatty acids (ΣMUFA) was higher in Marajó (RS—24.60%) and lower in Nova Timboteua (PS) (*p* < 0.05). Polyunsaturated fatty acids (ΣPUFA) were higher in feedlot animals (29.94%), and the lowest mean was observed in Lower Amazon buffaloes, in both periods (24.22; 24.22) (*p* < 0.05).

The sum of omega 6 fatty acids (Σn-6) was highest in confined water buffaloes (26.18), as was that of omega 3 (Σn-3) in animals raised in Nova Timboteua (RS—10.93) (*p* < 0.05). The highest sum of hypocholesterolemia (Σh) was observed in the livers of confined animals (48.82), and of hypercholesterolemia (ΣH) in Lower Amazon water buffaloes (RS—20.08), not differing from other pasture ecosystems and periods of the year, except Nova Timboteua (RS—15.87) and confinement (15.96) (*p* < 0.05).

### 3.4. Proportions and Indices of Fatty Acids

ΣDMA was higher in the liver tissues of animals from the Lower Amazon (DS—0.26) and feedlot (0.28) (*p* < 0.01), and the highest value of the ratio between polyunsaturated and saturated fatty acids (PUFA/SFA) was found in the livers of confined animals (0.70) (Table 7).

In the n-6/n-3 and hypo- and hypercholesterolemic (h/H) ratios, the feedlot animals again obtained the highest values (7.14 and 3.08), respectively (*p* < 0.01). AI was influenced only by location, with the highest mean in the Lower Amazon (2.13), followed by Nova Timboteua (1.69), Marajó (1.67) and feedlot (1.58) (*p* = 0.04).

The thrombogenicity index (TI) was influenced by the rearing system, with a lower average in the animals in the extensive system (0.23), against 0.32 in the confined animals (*p* < 0.01). Location also influenced TI, with the lowest mean observed in Nova Timboteua (0.20) (*p* = 0.04).

## 4. Discussion

### 4.1. Lipids, Cholesterol and Vitamins

The lowest total lipid content occurred in the tissues of buffalo raised in the ecosystem of Nova Timboteua (DS), which despite supplementation with wet brewery residue, had the lowest availability of forage mass in the pastures. The low availability and quality of the forage may have influenced the result, as the inclusion of supplementation occurred so that the animals did not have compromised performance in the face of the pasture situation. In the Amazon region, as with others in Brazil, the supplementation of animals on pasture is necessary in some periods of the year when it is verified that the availability and quality of the pasture are not meeting the requirements of the animals [14,32].

The same may explain the lower levels of cholesterol in animals from the island of Marajó (DS). Despite consuming forage in areas close to the floodplains, they were in a period of forage deficit in the pastures (Table 2). However, for humans, low consumption of foods rich in saturated fatty acids and cholesterol is recommended in order to prevent obesity, hypercholesterolemia, cardiovascular diseases, diabetes and the incidence of cancer [33,34]. Total cholesterol should be between 190 and 200 mg/dL of blood and its daily intake should not exceed 300 mg, according to nutritional recommendations from the World Health Organization [35]. Most meat cuts have cholesterol levels between 30 and 90 mg/100 g [36]; however, the viscera contain higher levels [37], as occurred in the present study.

The levels of α-tocopherol in all buffalo liver samples are considered high, especially when compared to the values found in meat [21,38]. The highest value, observed in the extensive pasture ecosystem in the Lower Amazon region (33.91 µg/g liver), could be attributed to the fact that the samples were collected at the end of the rainy season. During this period, when the rivers are low, there is an abundance of forage available (as pastures are flooded during the rainy season). Vitamin E, a major lipolytic antioxidant, plays a crucial role in both human and animal health and is primarily found in plasma, erythrocytes and the liver [39]. When consumed alongside vitamin A, it enhances the absorption of the latter, and its protective antioxidant effect on vitamin-A-carrying lipids may explain this increased absorption [40].

### 4.2. Fatty Acids

The biohydrogenation process may have influenced the summation of saturated fatty acid (ΣSFA) values. Thus, the livers of animals kept in extensive system obtained higher values compared to those in confinement [41]. The increase or decrease in acids in the tissue is related to the intensity of the biohydrogenation process and, therefore, influences the sum of fatty acids [42,43].

Among the SFA found (C12:0, C14:0, C15:0, C16:0, C17:0, C18:0 and C20:0), C12:0, C14:0 and C16:0 are hypercholesterolemic, and increase the concentration of lipids in human blood [44]. In contrast, oleic, linoleic and linolenic acids increase the number of hepatic LDL receptors and decrease their production and circulation in the plasma [45]. Stearic acid (C18:0), which represents between 10 and 20% of the fats produced by ruminants, does not appear to be cholesteremic and, when compared to palmitic acid, for example, reduces LDL cholesterol, but its effects on other risk markers for cardiometabolic outcomes have been poorly studied [46].

However, in general, consumption of SFA increases the risk of atherosclerotic diseases [47], with C18:0 being the only one that does not increase plasma cholesterol indices, acting (by adding to hepatic phospholipids and bile ducts, which affects micelles), in the reduction of cholesterol solubility [48,49,50].

MUFA (C14:1 c9, C16:1 c9, C17:1 c9, C18:1 c9 and C20:1 c11) were observed in the samples, have anticholesteremic characteristics, and their intake was inversely associated with total and cerebrovascular accident mortality [51,52]. In Marajó water buffaloes, the content of these fatty acids was significant (24.60%), and may have been influenced by the diet, as it occurred in the period of the greater availability of forage mass (RS).

All polyunsaturated fatty acids (PUFA) were also observed in the tissues, with significant values for the PUFA sums (ΣPUFA), reinforcing the nutritional quality of the liver, already mentioned in other works [20,53]. The highest value, which was observed in the livers of confined animals (29.94%), possibly occurred due to the diet, as it contained concentrate. PUFA can easily be increased through supplementation, and this can be observed in plasma and economically important tissues such as meat and liver [53]. PUFA have been listed as agents for the prevention and treatment of chronic neurological diseases, cancer, inflammatory diseases, obesity and diabetes mellitus [54,55,56].

The samples of food consumed by the animals were dried in an oven at 105 °C. This may have influenced the extraction of fatty acids, and explain the low levels of omega 3 and α-linolenic acid, when compared to other studies [45,53] (Table 1). They are essential fatty acids, as in addition to not being synthesized by the body, they promote health and its maintenance, and need to be part of the human diet [57].

Conjugated linoleic acid (C18:2 c9,t11—CLA), which is not synthesized by the human body but is of great biological importance [58], was observed in significant levels in the liver tissues of water buffaloes, mainly those reared in the Lower Amazon (RS) (1.63%), when there was a greater abundance of fodder. Its importance is due to its anticarcinogenic, antidiabetogenic (type 2 diabetes), antiatherogenic and immunomodulatory actions [59,60], which differ according to the type of isomer. The trans-10, cis-12 isomer is associated with combating the cell development of colon cancer [61], while CLA acts in breast cancer [62], and it was noticed in studies employed in breast cancer cells, that among the isomers cis-9 and cis-11, there are antitumor traits, possibly due to antiestrogenic attributes [63].

The superiority of confinement in the sum of ω 6 and the extensive system in the sum of omega 3 was possibly due to the fact that animals raised on pasture consumed more forage (rich in omega 3), while animals in confinement consumed more concentrates, which are rich in omega 6 [30,64]. Polyunsaturated fatty acids of the omega-6 family, which have pro-inflammatory characteristics, can stimulate mutations that cause cancerous diseases, while omega-3 fatty acids have anti-inflammatory characteristics [65,66,67], which may reduce the appearance of malignant tumors, including breast cancer [68,69].

### 4.3. Proportions of Fatty Acids

As for the nutritional values of fatty acids, the PUFA/SFA ratio, in all samples of liver tissue from water buffaloes, were greater than 0.45, the recommended level in the human diet [70], which indicates, yet again, that the consumption of liver is an opportune way to ensure the health of humans.

The n-6/n-3 ratio, with the exception of confined water buffaloes, was less than 4, the recommended value for the human diet [70]. However, this value is close to those found in other works with animals fed exclusively on pasture [64,71,72] and higher than those found in works evaluating buffalo meat [5,73]. The number found in the livers of feedlot animals (7.14) probably occurred due to feeding based on forage sorghum silage and concentrate. Confined animals, especially those fed high-concentrate diets, have high values for this ratio, which are even higher than those found in the present study [43,74,75].

The values of the hypocholesterolemic/hypercholesterolemic ratio (h/h) also demonstrate possible beneficial effects for human health when consuming this food. In meat products, values greater than 2 are recommended, as are those products that are characterized by fats of high nutritional quality and whose fatty acids promote the decrease in plasma cholesterol (hypocholesterolemic) and thus favor a reduction in the risk of cardiovascular diseases [76,77,78]. However, the values found are higher and/or compatible with the meat of several evaluated species [79].

The thrombogenicity index (TI) considers C14:0, C16:0 and C18:0 acids as thrombogenic, and monounsaturated and polyunsaturated fatty acids (omega 6 and omega 3) as antithrombogenic [31,80]. Although there is no threshold value, the TI should be as low as possible, demonstrating a good amount of fatty acids that promote health. When lower, it also shows a greater amount of antiatherogenic fatty acids, and consequently, a greater ability to prevent coronary heart disease [81,82]. The AI, which assesses the capacity for plaque formation in blood vessels [83], was above the maximum recommended value for meat of 1.0 [84].

Diets rich in n-3 PUFA, among other benefits, reduce the risk of thrombosis, atherosclerosis, heart disease and neurological disorders, and improve learning and visual acuity [80,85]. Because of this and their anti-inflammatory ability, they have been the focus of several studies that investigated the fight against the spread of infections [55,86,87]. However, excessive consumption has undesirable effects, as it makes it impossible to respond to infection and causes mutations in blood clotting, with a predisposition to hemorrhage [88,89].

In the context of future prospects, the results of this study have significant implications for buffalo farming in the Amazon region. Buffalo producers can benefit by carefully considering the ecosystems and seasons in their management and diet systems in order to improve the quality of the meat produced. Furthermore, these findings underscore the need for additional research to better understand the relationships between diet, environment and nutritional composition, which can lead to more sustainable farming practices and the production of healthier food for human consumption. Therefore, the study sheds light on the importance of a holistic and sustainable approach to buffalo meat production in the Amazon, aiming for both product quality and the preservation of local ecosystems.

## 5. Conclusions

The composition of buffalo liver tissues, including cholesterol, tocopherols, β-carotene and fatty acid profiles, is significantly influenced by the specific ecosystems of the Amazon and the distinct dry and rainy seasons of the year. Total lipid content is notably impacted by geographic location and time of year, with statistically significant differences. Furthermore, variables such as total cholesterol, α-tocopherol and γ-tocopherol show significant variations based on factors such as extensive versus intensive ecosystems, location, time periods and the interaction between location and time of year. Thus, pasture ecosystems and seasons influence the nutritional values of buffalo liver tissue but do not compromise nutritional quality. The liver is a food with great nutritional potential, but consumption must be guided by a professional, paying attention to some values (PUFA/SFA; h/H; n-6/n-3; AI and IT).

## Figures and Tables

**Table 1:** Chemical composition of diets from water buffaloes managed on different ecossystems on eastern amazon.

Items	Lower Amazon		Marajó		Nova Timboteua		Intensive	
	DS	RS	DS	RS	DS	RS	Forage	RUC
**Chemical composition (% DM)**								
DM	24	24.1	23.9	18.3	23.3	26.1	24.7	39
OM	91.5	91	89.5	84.9	91.8	96.9	95.4	94.8
CP	7.9	8.7	7.6	8.9	7.7	8.3	9.4	8.3
NDF	73.2	79.1	68.7	70.9	75.7	55.7	69	54.4
NFC	9.1	1.8	11.8	3.1	6.3	5.2	13.9	29.6
ADF	44.9	54.9	40.1	43.9	55.8	22.6	44.5	38.1
TDN	51.6	42.2	56.1	52.5	41.4	72.5	51.9	57.9
Ash	8.5	9	10.6	15.1	8.2	3.1	6.2	5.2
TL (mg/g diet)	4.84	4.71	5.17	4.04	5.07	-	2.88	9.77
α-Tocopherol (µg/g DM)	7.76	4.1	5.33	6.77	6.75	23.7	9.4	17
γ-Tocopherol (µg/g DM)	0.9	0.12	0.13	0.15	0.16	3.27	0.44	0.59
γ-Tocotrienol (µg/g DM)	0.71	0.98	1.65	2	1.9	27.54	2.16	nd
**Fatty acid composition (% /total FA)**								
12:00	3.03	1.85	1.84	1.03	2.45	0.03	1.03	0.11
14:00	3.57	2.81	3.35	3.33	2.63	0.38	3.33	0.18
14:1 c9	0.86	1.73	2.27	1.41	0.38	0	1.41	0.07
15:00	1.05	0.96	0.76	0.99	0.55	0.11	0.99	0.13
16:00	37.52	40.82	36.6	50.63	32.61	25.3	50.63	18.5
16:1 c9	2.11	1.96	1.28	1.09	0.72	0.18	1.09	0.34
17:00	3.87	3.27	6.38	4.26	2.3	0.15	4.26	0.44
17:1 c9	1.52	1.23	0.66	2.05	0.29	0.04	2.05	0.07
18:00	11.77	6.93	13.93	9.61	6.63	1.52	9.61	2.59
18:1 c9	12.49	7.7	11.33	5.45	20.53	9.89	5.45	33.63
18:1 c11	1.96	1.54	2.1	1.6	1.82	0.85	1.6	1.77
18:2n6	10.35	14.25	9.45	7.37	21.17	55.25	7.37	38.18
18:3n6	0	0	0.61	0	0	0	0	0
18:3 n-3	5.71	12.71	4.01	7.37	5.81	5.56	7.37	3.38
20:00	3.92	2.23	5.43	3.81	1.66	0.25	3.81	0.41
20:1 c11	0	0	0	0	0.44	0.5	0	0.22

Diets (*n* = 3) fed to Murrah × Mediterranean crossbred water buffaloes that were extensively (three production system types × dry (DS) and rainy (RS) seasonal periods) or intensivelly reared. Intensive = intensive breeding system (confined). For = fodder; WBR = wet brewery residue; nd, not detected; DM, dry matter; OM, organic matter; CP, crude protein; NDF, neutral detergent fiber; NFC, non-fiber carbohydrates; ADF, acid detergent fiber; TDN, total digestible nutrients; TL, total lipids. BWW-Brewery Wet Waste.

**Table 2:** Soil characteristics from water buffaloes managed on different ecossystems on eastern amazon.

Items	Lower Amazon		Marajó		Nova Timboteua	
	DS	RS	DS	RS	DS	RS
C	13.9	11.6	20.8	7.5	7.3	26.8
P	3.0	2.0	3.0	3.0	9.0	11.0
K	170.0	14.0	197.0	589.0	17.0	105
Na	6.0	4.0	1366	1054	6.0	58.0
Al	0.1	0.2	1.1	0.8	0.3	2.1
Ca	1.5	0.8	2.7	4.0	0.7	0.7
Mg	0.8	0.4	12.5	14.3	0.3	1.6
Ca + Mg	2.3	1.2	15.2	18.3	1.1	2.3
**Other characteristics**						
OM	24.0	20.0	36.0	12.9	12.6	46.2
pH	4.5	4.5	4.2	5.0	4.4	4.2
H + Al	3.8	3.5	9.1	6.15	3.5	8.7
CEP Total	6.5	4.7	30.7	30.6	4.6	11.5
CEP effective	2.9	1.5	22.7	25.2	1.5	4.9
Saturation V%	42.1	26.8	70.4	79.9	25.5	24.7
Saturation M%	4.2	16.0	5.0	3.0	22.4	42.3

C = carbon; P = phosphorus; K = potassium; Na = sodium; Al = aluminum; Ca = calcium; Mg = magnesium; OM = organic matter; H = hydrogen; CEP = cation exchange capacity.

**Table 3:** Formulas used in study.

n-6/n-3PUFA = (18:2 n-6 + 18:3 n-6 + 20:2 n-6 + 20:3 n-6 + 20:4 n-6 + 22:4 n-6)/(18:3 n-3 + 20:5 n-3 + 22:5 n-3 + 22:6 n-3)
PUFA/SFA = (PUFA n-6 +PUFA n-3)/(4:0 + 6:0 + 8:0 + 10:0 + 12:0 + 14:0 + 15:0 + 16:0 + 17:0 + 18:0 + 20:0)
h/H = (18:1 c9 + PUFA n-6 + PUFA n-3)/(12:0 + 14:0 + 16:0)
IA (index of atherogenicity) = [(12:0) + (4 × 14:0) + (16:0)] [(ΣPUFA n-6 + ΣPUFA n-3) + ΣMUFA)]
IT (index of thrombogenicity) = [(14:0) + (16:0) + (18:0)]/[(0.5 × ΣMUFA) + (0.5 × ΣPUFA n-6) + (3 × ΣPUFA n-3) + (ΣPUFA n-3 × ΣPUFA n-6)]

**Table 4:** Fat-soluble compounds of livers of crossbred water buffaloes (*n* = 12) raised extensively in three types of production systems, during dry (DS) and rainy (RS) seasonal periods and in feedlot, in Eastern Amazonia (Brazil).

Items	Extensive Systems						Intensive	SEM	*p*-Values			
	Lower Amazon		Marajó		Timboteua				E vs. I	Local (L) ^1^	Period (P) ^2^	L × P
	DS	RS	DS	RS	DS	RS						
**mg/g liver**												
TL	40.72 a	41.44 a	32.34 b	35.08 ab	31.21 b	36.95 ab	32.61 b	1.54	0.06	<0.01 (S > M = N)	0.02	0.27
TC	2.57 a	2.34 ab	1.82 d	2.31 abc	2.00 bcd	2.49 a	1.97 cd	0.08	<0.01	<0.01 (S > M = N)	<0.01	<0.01
**µg/g liver**												
α-tocoferol	12.90 cd	33.91 a	8.50 d	14.60 cd	22.29 bc	29.99 ab	12.49 cd	2.63	<0.01	<0.01 (S = N > M)	<0.01	0.02
γ-tocoferol	0.31 d	1.17 a	0.30 d	0.39 d	0.45 cd	0.65 bc	0.74 b	0.05	0	<0.01 (S > N > M)	<0.01	<0.01
γ-tocotrienol	0.84 b	nd	0.97 ab	1.26 ab	0.97 ab	1.12 ab	1.49 a	-	-	0.613	0.08	0.59

Livers of Murrah × Mediterranean crossbred water buffaloes (*n* = 12) raised extensively in three types of production systems, during dry (DS) and rainy (RS) seasonal periods and feedlot. TL, total lipids; TC, total cholesterol; ^1^ production systems; ^2^ season periods; (S = M < N) indicates the difference among the location (Santarém, Marajó and Nova Timboteua). Values with different letters (a, b, c, d) within a line differ significantly at *p* < 0.05; nd, undetected.

**Table 5:** Fatty acid composition of livers of crossbred water buffaloes (*n* = 12) raised extensively in three types of production systems, dry (DS) and rainy (RS) seasonal periods, and in feedlots.

Items	Extensive						Intensive (I)	SEM	*p*-Values			
	Lower Amazon		Marajó		Timboteua				E vs. I	Local (L) ^1^	Period (P) ^2^	L × P
	DS	RS	DS	RS	DS	RS						
12:00	0.15 ab	0.10 c	0.11 bc	0.10 c	0.16 a	0.11 c	0.07 c	0.01	<0.01	0.07	<0.01 (DP > RP)	0.06
14:00	0.34 ab	0.47 a	0.28 b	0.32 ab	0.33 ab	0.28 ab	0.24 ab	0.04	0.04	0.05 (S = M = N)	0.34	0.19
14:1 *c*9	0.06 ab	0.03 bc	0.03 c	0.04 abc	0.03 c	0.02 c	0.06 a	0.01	<0.01	<0.01	0.07	0.03
15:00	0.38 bcd	0.51 ab	0.31 d	0.33 cd	0.61 a	0.47 abc	0.23 d	0.04	<0.01	<0.01	0.82	0.01
16:00	17.66 ab	19.52 a	17.88 ab	18.61 ab	17.60 ab	15.49 b	15.58 b	0.69	<0.01	0.03	0.80	0.04
16:1 *c*9	0.87 abc	1.05 a	0.76 c	0.99 abc	1.02 ab	1.06 a	0.79 bc	0.06	<0.01	0.04 (N > M = S)	<0.01 (DP < RP)	0.30
17:00	3.78 a	2.37 b	2.11 b	2.15 b	2.38 b	2.79 b	0.88 c	0.15	<0.01	<0.01	0.03	<0.01
17:1 *c*9	0.40 a	0.43 a	0.38 a	0.41 a	0.36 ab	0.41 a	0.28 b	0.02	<0.01	0.50	0.06	0.91
18:00	25.24 ab	24.03 ab	24.87 ab	23.04 ab	25.24 ab	25.24 ab	25.60 a	0.53	0.09	0.11	0.04 (DP > RP)	0.31
18:1(1)	3.21 bcd	3.55 b	2.99 cd	3.39 bc	4.20 a	4.10 a	2.91 d	0.11	<0.01	<0.01 (S = M < N)	0.04	0.10
18:1 *c*9	18.15 ab	18.67 a	18.30 ab	19.69 a	14.69 c	15.38 bc	18.88 a	0.73	0.08	<0.01 (S = M > N)	0.19	0.84
18:2 *n*-6	7.43 b	6.98 b	8.52 b	6.82 b	8.10 b	7.30 b	12.06 a	0.41	<0.01	0.46	0.01 (DP > RP)	0.34
18:3 *n*-6	0.18 b	0.18 b	0.17 b	0.19 b	0.18 b	0.19 b	0.37 a	0.02	<0.01	0.84	0.67	0.93
18:3 *n*-3	1.67 ab	1.76 ab	1.57 ab	1.54 b	1.55 ab	1.98 a	0.47 c	0.09	<0.01	0.14	0.07	0.12
18:2 *c*9,*t*11	0.82 de	1.63 a	1.05 cd	1.52 ab	1.08 cd	1.19 bc	0.57 e	0.08	<0.01	0.25	<0.01	<0.01
20:00	0.12 ab	0.13 ab	0.12 b	0.15 a	0.11 bc	0.10 bc	0.08 c	0.01	<0.01	<0.01	0.16	0.02
20:1 *c*11	0.09 a	0.07 ab	0.05 b	0.08 a	0.08 a	0.08 a	0.09 a	0.01	<0.01	0.06	0.41	0.01
20:2 *n*-6	0.61	0.69	0.52	0.75	0.65	0.84	0.79	0.07	0.15	0.33	0.01 (DP < RP)	0.59
20:3 *n*-6	1.82 bc	1.60 c	1.69 c	1.55 c	2.48 b	2.38 b	4.40 a	0.15	<0.01	<0.01 (S = M < N)	0.18	0.92
20:4 *n*-6	5.93 b	5.85 b	6.96 b	6.23 b	6.39 b	5.91 b	8.55 a	0.39	<0.01	0.24	0.22	0.74
20:5 *n*-3	2.53 a	2.86 a	2.93 a	3.24 a	2.80 a	3.53 a	0.81 b	0.22	<0.001	0.14	0.03 (DP < RP)	0.65
22:5 *n*-3	3.47 a	3.64 a	4.42 a	4.24 a	4.16 a	4.75 a	2.07 b	-	-	0.01 (S < M = N)	0.45	0.49
22:6 *n*-3	0.58 bc	0.66 ab	0.86 a	0.80 ab	0.66 ab	0.67 ab	0.41 b	0.05	<0.01	<0.01 (M > S = N)	0.83	0.48
DMA16:0	0.14 a	0.06 b	0.09 ab	0.07 b	0.08 ab	0.04 b	0.14 a	0.02	<0.01	0.03 (S > N = M)	<0.01 (DP > RP)	0.17
DMA18:0	0.10 ab	0.05 c	0.07 abc	0.06 bc	0.07 abc	0.04 c	0.11 a	0.01	<0.01	0.24	<0.01 (DP > RP)	0.19
DMA18:1	0.02 ac	0.01 b	0.02 bc	0.01 c	0.02 ab	0.01 bc	0.03 a	0.01	<0.01	0.01 (S > M = N)	<0.01	0.36
Other	5.08 bc	4.73 bcd	3.99 cd	5.20 bc	6.06 ab	6.86 a	3.46 d	0.34	<0.01	<0.01 (N > S = M)	0.08	0.10

SEM, standard error of the mean; ^1^ production systems; ^2^ season periods; (S > M = N), (S = M < N) and (M > S = N) indicate the differences among the locations (Santarém, Marajó and Nova Timboteua); (DS > RS) and (DS < RS) indicate the differences between the periods (dry and rainy). 12:0, lauric; 14:0, myristic; 14:1 c9, myristoleic; 15:0, pentadecylic; 16:0, palmitic; 16:1 c9, palmitoleic; 17:0, margaric; 17:1 c9, heptadecaenoic; 18:0, stearic; 18:1(1) = sum of 18:1cis and 18:1trans; 8:1 c9, oleic; 18:2 n-6, linoleic acid; 18:3 n-6, γ-linolenic; 18:3 n-3, α-linolenic; 18:2 c9,t11, conjugated linoleic—CLA; 20:0, arachidic; 20:1 c11, eicosaenoic; 20:2 n-6, eicosadienoic; 20:3 n-6, dihomo-gamma-linoleic; 20:4 n-6, arachidonic; 20:5 n-3, eicosapentaenoic; 22:5 n-3, docosapentaenoic; 22:6 n-3, docosahexaenoic; DMA, dimethyl acetals; other, non-identified fatty acids. a, b, c, d: values with different superscripts within a row differ significantly at *p* < 0.05.

**Table 6:** Partial sums of fatty acids of livers of crossbred water buffaloes (*n* = 12) raised extensively in three types of production systems during dry (DS) and rainy (RS) seasonal periods and in feedlot.

Items	Extensive						Intensive (I)	SEM	*p*-Values			
	Lower Amazon		Marajó		Timboteua				E vs. I	Local (L) ^1^	Period (P) ^2^	L × P
	DS	RS	DS	RS	DS	RS						
ΣSFA	47.67 a	47.12 ab	45.69 ab	44.71 abc	46.42 ab	44.48 bc	42.75 c	0.68	<0.01	0.01 (S > M = N)	0.07	0.66
ΣMUFA	22.77 ab	23.81 ab	22.50 ab	24.60 a	20.37 b	21.04 ab	23.02 ab	0.81	0.57	<0.01 (S = M > N)	0.09	0.70
ΣPUFA	24.22 b	24.22 b	27.64 ab	25.36 ab	26.98 ab	27.54 ab	29.94 a	1.08	<0.01	0.04 (N > S = M)	0.56	0.46
Σ*n*-6	15.97 b	15.30 b	17.86 b	15.54 b	17.80 b	16.61 b	26.18 a	0.68	<0.01	0.12	0.03 (DP > RP)	0.52
Σ*n*-3	8.25 b	8.93 ab	9.78 ab	9.82 ab	9.18 ab	10.93 a	3.76 c	0.56	<0.01	0.05 (S = M = N)	0.11	0.39
Σh	42.37 cd	42.89 bcd	45.94 ab	45.05 bc	41.67 d	42.92 bcd	48.82 a	0.74	<0.01	<0.01 (M > S = N)	0.67	0.42
ΣH	18.15 ab	20.08 a	18.28 ab	19.03 ab	18.09 ab	15.87 b	15.96 b	0.73	<0.01	0.03	0.81	0.04
ΣDMA	0.26 a	0.12 b	0.18 ab	0.14 b	0.17 ab	0.09 b	0.28 a	0.03	<0.01	0.08	<0.01 (DP > B)	0.21

SEM, standard error of the mean; ^1^ production systems; ^2^ season periods; (S = M < N) indicate the differences among the locations (Santarém, Marajó and Nova Timboteua); (DS > RS) indicates the differences between the periods (dry and rainy); SFA, saturated fatty acids; MUFA, monounsaturated fatty acids; PUFA, polyunsaturated fatty acids; ΣSFA = sum of 12:0, 14:0, 15:0, 16:0, 17:0, 18:0 and 20:0; ΣMUFA = sum of 14:1 c9, 16:1 c9, 17:1 c9, 18:1 c9, 18:1 and 20:1 c11; ΣPUFA = sum of 18:2 n-6, 18:3 n-6, 18:3 n-3, 20:2 n-6, 20:3 n-6, 20:4 n-6, 20:5 n-3, 22:5 n-3 and 22:6 n-3; Σ n-6 = sum of 18:2 n-6, 18:3 n-6, 20:2 n-6, 20:3 n-6 and 20:4 n-6; Σn-3 = sum of 18:3 n-3, 20:5 n-3, 22:5 n-3 and 22:6 n-3; ΣDMA = DMA 16:0, 18:0 and 18:1. a, b, c, d: values with different superscripts within a row differ significantly at *p* < 0.05.

**Table 7:** Nutritional indices of fatty acids of livers of crossbred water buffaloes (*n* = 12) raised extensively in three types of production systems, during dry (DS) and rainy (RS) seasonal periods and in feedlot.

Items	Extensive						Intensive	SEM	*p*-Values			
	Lower Amazon		Marajó		Timboteua				E * I	Local (L) ^1^	Period (P) ^2^	L × P
	DS	RS	DS	RS	DS	RS						
PUFA/SFA	0.52 b	0.52 b	0.61 ab	0.57 b	0.59 ab	0.62 ab	0.70 a	0.03	<0.01	0.02 (N > S = M)	0.92	0.50
*n*-6/*n*-3	2.23 b	1.76 b	1.86 b	1.61 b	2.07 b	1.53 b	7.14 a	0.19	<0.01	0.22	<0.01 (DP > RP)	0.63
h/H	2.43 b	2.18 b	2.58 b	2.40 b	2.39 b	2.73 b	3.08 a	0.12	<0.01	0.16	0.79	0.07
IA	1.89 ab	2.37 a	1.59 ab	1.74 ab	1.85 ab	1.53 b	1.58 ab	0.19	0.23	0.04 (S = M = N)	0.53	0.17
IT	0.30 ab	0.26 ab	0.22 ab	0.22 ab	0.22 ab	0.18 b	0.32 a	0.03	<0.01	0.04 (S = M = N)	0.28	0.68

SEM, standard error of the mean; ^1^ production systems; ^2^ season periods; (S = M < N) indicate the differences among the locations (Santarém, Marajó and Nova Timboteua); (DS > RS) indicates the differences between the periods (dry and rainy); PUFA/SFA = (Σ18:2 n-6, 18:3 n-6, 18:3 n-3, 20:2 n-6, 20:3 n-6, 20:4 n-6, 20:5 n-3, 22:5 n-3 and 22:6 n-3)/(Σ12:0, 14:0, 15:0, 16:0, 17:0, 18:0 and 20:0); n-6/n-3 = (Σ18:2 n-6, 18:3 n-6, 20:2 n-6, 20:3 n-6 and 20:4 n-6)/(Σ18:3 n-3, 20:5 n-3, 22:5 n-3 and 22:6 n-3); h/H = hypocholesterolemic/hypercholesterolemic ratio = (Σ18:1 c9, 18:2 n-6, 18:3 n-6, 18:3 n-3, 20:2 n-6, 20:3 n-6, 20:4 n-6, 20:5 n-3 and 22:5 n-3)/(Σ12:0, 14:0 and 16:0); IA = index of atherogenic = [(12:0) + (4 × 14:0) + (16:0)]/[(Σ18:2 n-6, 18:3 n-6, 20:2 n-6, 20:3 n-6 e 20:4 n-6 + 18:3 n-3, 20:5 n-3, 22:5 n-3 and 22:6 n-3) + (Σ14:1 c9, 16:1 c9, 17:1 c9, 18:1 c9, 18:1 and 20:1 c11)]; IT = index of thrombogenic = [Σ14:0, 16:0 and 18:0)]/[0.5 × (14:1 c9 + 16:1 c9 + 17:1 c9 + 18:1 c9 + 18:1 + 20:1 c11)] + [(0.5 × (18:2 n-6 + 18:3 n-6 + 20:2 n-6 + 20:3 n-6 + 20:4 n-6)] + [3 × (18:3 n-3 + 20:5 n-3 + 22:5 n-3 + 22:6 n-3)] + (Σ18:3 n-3, 20:5 n-3, 22:5 n-3 and 22:6 n-3 × Σ18:2 n-6, 18:3 n-6, 20:2 n-6, 20:3 n-6 and 20:4 n-6)]; a, b: values with different superscripts within a row differ significantly at *p* < 0.05.

## Data Availability

The data presented in this study are available upon reasonable request from the corresponding author.

## References

[1] Maurano L.E.P., Escada M.I.S. Comparação dos dados produzidos pelo PRODES versus dados do MapBiomas para o bioma Amazônia. Proceedings of the Simpósio Brasileiro de Sensoriamento Remoto.

[2] IBGE—Instituto Brasileiro de Geografia e Estatística (2022). Diretoria de Pesquisas, Coordenação de Agropecuária. Pesquisa da Pecuária Municipal. https://sidra.ibge.gov.br/tabela/3939#resultado.

[3] Araújo N.N.A., dos Santos Junior H.B., Araújo E.A.A., Souza F.P., de Andrade V.M.S., da Silva Carneiro F., de Assis Oliveira F. (2021). Estoque de nutrientes e retenção hídrica da liteira em três ecossistemas florestais da Amazônia oriental brasileira. Res. Soc. Dev..

[4] Ávila J.V.C., Clement C.R., Junqueira A.B., Ticktin T., Steward A.M. (2021). Adaptive management strategies of local communities in two Amazonian floodplain ecosystems in the face of extreme climate events. J. Ethnobiol..

[5] Silva C.C.G., Domingos-Lopes M.F.P., Coelho M.C., Rego O.A., Rosa H.J.D. (2022). The effect of corn supplementation on α-tocopherol concentration and oxidative stability in three muscles of grass-fed Holstein bulls. Anim. Prod. Sci..

[6] Rodrigues L.S., da Silva J.A.R., Lourenço-Júnior J.D.B., Silva A.G.M.E., de Almeida A.M., Mourato M.P., de Castro V.C.G., Bezerra A.S., da Silva W.C., Prates J.A.M. (2023). Muscle mineral profile of water buffaloes (*Bubalus bubalis*) reared in different production in systems of the Brazilian Eastern Amazon. Front. Vet. Sci..

[7] Rodrigues L.S., da Silva J.A.R., Lourenço-Júnior J.D.B., Silva A.G.M.E., de Almeida A.M., Mourato M.P., de Castro V.C.G., da Silva W.C., Prates J.A.M. (2023). Mineral Content of Liver of Buffaloes (*Bubalus bubalis*) Reared in Different Ecosystems in the Eastern Amazon. Animals.

[8] Silva J.A.R.D., Rodrigues L.S., Lourenço-Júnior J.D.B., Alfaia C.M., Costa M.M., Castro V.C.G.D., Bezerra A.S., Almeida A.M.D., Prates J.A.M. (2022). Total Lipids, Fatty Acid Composition, Total Cholesterol and Lipid-Soluble Antioxidant Vitamins in the longissimus lumborum Muscle of Water Buffalo (*Bubalus bubalis*) from Different Production Systems of the Brazilian Eastern Amazon. Animals.

[9] Valente G.F., Ferraz G.A.E.S., Santana L.S., Ferraz P.F.P., Mariano D.D.C., dos Santos C.M., Okumura R.S., Simonini S., Barbari M., Rossi G. (2022). Mapping Soil and Pasture Attributes for Buffalo Management through Remote Sensing and Geostatistics in Amazon Biome. Animals.

[10] Garcia A.R., Silva L.K.X., Barros D.V., Junior J.D.B.L., Martorano L.G., Lisboa L.S.S., da Silva J.A.R., de Sousa J.S., da Silva A.O.A. (2023). Key points for the thermal comfort of water buffaloes in Eastern Amazon. Ciência Rural..

[11] Barbosa J.D., Brito M.F., Lins A.D.M.C., Barbosa C.C., da Costa P.S.C., Duarte M.D., Ferreira T.T.A., Silveira N.D.S.E.S., Oliveira C.M.C., Salvarani F.M. (2023). Proximal and Distal Vagal Indigestion in Buffaloes (*Bubalus bubalis*) in the Amazon Biome. Vet. Sci..

[12] IBGE Mapa de Biomas e de Vegetação. https://ww2.ibge.gov.br/.

[13] Neto B., Oliveira C., Duarte D., Albernaz T., Riet-Correa G., Riet-Correa F. (2007). Phosphorus deficiency in buffaloes in the state of Pará, Northern Brazil. Ital. J. Anim. Sci..

[14] da Silva W.C., Araújo L.J.S., Silva L.K.X., Reis A.D.S.B. (2021). Lesões de pele diagnósticadas em búfalos (*Bubalis bubalis*) na região do baixa amazonas. Rev. Bras. Ciência Veterinária.

[15] Mota-Rojas D., Titto C.G., Orihuela A., Martínez-Burnes J., Gómez-Prado J., Torres-Bernal F., Flores-Padilla K., Carvajal-De la Fuente V., Wang D. (2021). Physiological and behavioral mechanisms of thermoregulation in mammals. Animals.

[16] da Silva J.A.R., de Andrade Pantoja M.H., da Silva W.C., de Almeida J.C.F., Noronha R.D.P.P., Barbosa A.V.C., Júnior J.D.B.L. (2022). Thermoregulatory reactions of female buffaloes raised in the sun and in the shade, in the climatic conditions of the rainy season of the Island of Marajó, Pará, Brazil. Front. Vet. Sci..

[17] da Silva W.C., da Silva J.A.R., Camargo-Júnior R.N.C., da Silva B.R., dos Santos M.R.P., Viana R.B., Silva A.G.M.E., da Silva C.M.G., Lourenço-Júnior J.D.B. (2023). Animal welfare and effects of per-female stress on male and cattle reproduction—A review. Front. Vet. Sci..

[18] Bertoni A., Álvarez-Macías A., Mota-Rojas D., Dávalos J.L., Minervino A.H.H. (2021). Dual-purpose water buffalo production systems in tropical latin America: Bases for a sustainable model. Animals.

[19] Athaíde L.G., Joset W.C.L., de Almeida J.C.F., Pantoja M.H.D.A., Noronha R.D.P.P., Bezerra A.S., Barbosa A.V.C., Martorano L.G., da Silva J.A.R., Júnior J.D.B.L. (2020). Thermoregulatory and behavioral responses of buffaloes with and without direct sun exposure during abnormal environmental condition in Marajó Island, Pará, Brazil. Front. Vet. Sci..

[20] Biel W., Czerniawska-Piątkowska E., Kowalczyk A. (2019). Offal chemical composition from veal, beef, and lamb maintained in organic production systems. Animals.

[21] Fűrész A., Penksza K., Sipos L., Turcsányi-Járdi I., Szentes S., Fintha G., Penksza P., Viszló L., Szalai F., Wagenhoffer Z. (2023). Examination of the Effects of Domestic Water Buffalo (*Bubalus bubalis*) Grazing on Wetland and Dry Grassland Habitats. Plants.

[22] Detmann E., Souza M.A., Valadares Filho S.C., Queiroz A.C., Berchielli T.T., Saliba E.O.S. (2012). Métodos para Análise de Alimentos—INCT—Ciência Animal.

[23] Sniffen C.J., O’Connor J.D., Van Soest P.J., Fox D.G., Russell J.B. (1992). Net carbohydrate and protein system for evaluating cattle diets: II. Carbohydrate and protein availability. J. Anim. Sci..

[24] Prates J.A.M., Quaresma M.A., Bessa R.J.B., Fontes C.M.G.A., Alfaia C.P.M. (2006). Simultaneous HPLC quantification of total cholesterol, tocopherols and β-carotene in Barrosã—PDO veal. Food Chem..

[25] Folch J., Lees M., Stanley G.H.S. (1957). A simple method for the isolation and purification of total lipids from animal tissues. J. Biol. Chem..

[26] Carlson L.A. (1985). Extraction of lipids from human whole serum and lipoproteins and from rat liver tissue with methylene chloride-methanol: A comparison with extraction with chloroform-methanol. Clin. Chim. Acta.

[27] Pestana J., Puerta B., Santos H., Madeira M., Alfaia C., Lopes P., Pinto R., Lemos J., Fontes C., Lordelo M. (2020). Impact of dietary incorporation of Spirulina (*Arthrospira platensis*) and exogenous enzymes on broiler performance, carcass traits, and meat quality. Poult. Sci..

[28] Alves S.P., Bessa R.J.B. (2009). Comparison of two gas–liquid chromatographic columns for the analysis of fatty acids in ruminant meat. J. Chromatogr. A.

[29] British Department of Health (1994). Nutritional Aspects of Cardiovascular Disease.

[30] Enser M., Hallett K.G., Hewett B., Fursey G.A.J., Wood J.D., Harrington G. (1998). Fatty acid content and composition of UK beef and lamb muscle in relation to production system and implications for human nutrition. Meat Sci..

[31] Ulbricht T.L.V., Southgate D.A.T. (1991). Coronary heart disease: Seven dietary factors. Lancet.

[32] Galvão L.T.O., Reis G.C., Silva C.C., Pinto A.S., Santos D.M., Lima E.M., Gomes D.I., Oliveira L.R.S., Alves K.S., Santos P.M. (2020). Performance of lactating buffaloes in pasture supplemented with palm-kernel cake. Anim. Prod. Sci..

[33] Husson M.O., Ley D., Céline P., Hueso T., Desseyn J.L., Gottrand F. (2016). Modulation of host defence against bacterial and viral infections by omega-3 polyunsaturated fatty acids. J. Infect..

[34] Nassar Z.D., Aref A.T., Miladinovic D., Mah C.Y., Raj G.V., Hoy A.J., Butler L.M. (2018). Peri-prostatic adipose tissue: The metabolic microenvironment of prostate cancer. BJU Int..

[35] Who J., Consultation F.E. (2003). Diet, Nutrition and the Prevention of Chronic Diseases.

[36] Valsta L.M., Tapanainen H., Mannisto S. (2005). Meat fats in nutrition. Meat Sci..

[37] Pratiwi N.M., Murray P.J., Taylor D.G. (2006). Total cholesterol concentrations of the muscles in castrated boer goats. Small Rumin. Res..

[38] Jerónimo E., Soldado D., Sengo S., Francisco A., Fernandes F., Portugal A.P., Alves S.P., Santos-Silva J., Bessa R.J. (2020). Increasing the α-tocopherol content and lipid oxidative stability of meat through dietary *Cistus ladanifer* L. in lamb fed increasing levels of polyunsaturated fatty acid rich vegetable oils. Meat Sci..

[39] Suzuki H., Kume A., Herbas M. (2018). Potential of Vitamin E Deficiency, Induced by Inhibition of α-Tocopherol Efflux, in Murine Malaria Infection. Int. J. Mol. Sci..

[40] Godswill A.G., Somtochukwu I.V., Ikechukwu A.O., Kate E.C. (2020). Health benefits of micronutrients (vitamins and minerals) and their associated deficiency diseases: A systematic review. Int. J. Food Sci..

[41] De Smet S., Raes K., Demeyer D. (2004). Meat fatty acid composition as affected by fatness and genetic factors: A review. Anim. Res..

[42] Wood J.D., Richardson R.I., Nute G.R., Fisher A.V., Campo M.M., Kasapidou E., Sheard P.R., Enser M. (2004). Effects of fatty acids on meat quality: A review. Meat Sci..

[43] Junior P.C.G.D., dos Santos I.J., da Silva A.L., de Assis R.G., Vicente A.C.S., Carlis M.S., Soares L.C., Comelli J.H., Biava J.S., Araujo R.C. (2023). Essential oil from *Arnica montana* on feedlot performance, ingestive behavior, carcass characteristics, rumen morphometrics characteristics and meat fatty acids profile of lambs. Small Rumin. Res..

[44] Paszczyk B., Łuczyńska J. (2020). The comparison of fatty acid composition and lipid quality indices in hard cow, sheep, and goat cheeses. Foods.

[45] Teh H.E., Yokoyama W.H., German J.B., McHugh T.H., Pan Z. (2019). Hypocholesterolemic effects of expeller-pressed and solvent-extracted fruit seed oils and defatted pomegranate seed meals. J. Agric. Food Chem..

[46] Van Rooijen M.A., Mensink R.P. (2020). Palmitic acid versus stearic acid: Effects of interesterification and intakes on cardiometabolic risk markers—A systematic review. Nutrients.

[47] Mensink R.P. (2005). Effects of stearic acid on plasma lipid and lipoproteins in humans. Lipids.

[48] Hunter J.E., Zhang J., Kris-Etherton P.M. (2010). Cardiovascular disease risk of dietary stearic acid compared with trans, other saturated, and unsaturated fatty acids: A systematic review. Am. J. Clin. Nutr..

[49] Scollan N.D., Dannenberger D., Nuernberg K., Richardson I., MacKintosh S., Hocquette J.F., Moloney A.P. (2014). Enhancing the nutritional and health value of beef lipids and their relationship with meat quality. Meat Sci..

[50] Jesch E.D., Carr T.P. (2017). Food ingredients that inhibit cholesterol absorption. Prev. Nutr. Food Sci..

[51] Kim Y., Je Y., Giovannucci E.L. (2021). Association between dietary fat intake and mortality from all-causes, cardiovascular disease, and cancer: A systematic review and meta-analysis of prospective cohort studies. Clin. Nutr..

[52] Mazidi M., Mikhailidis D.P., Sattar N., Toth P.P., Judd S., Blaha M.J., Hernandez A.V., Penson P.E., Banach M. (2020). Association of types of dietary fats and all-cause and cause-specific mortality: A prospective cohort study and meta-analysis of prospective studies with 1,164,029 participants. Clin. Nutr..

[53] Hennessy A.A., Kenny D.A., Byrne C.J., Childs S., Ross R.P., Devery R., Stanton C. (2021). Fatty acid concentration of plasma, muscle, adipose and liver from beef heifers fed an encapsulated n-3 polyunsaturated fatty acid supplement. Animal.

[54] Fiolet T., Srour B., Sellem L., Kesse-Guyot E., Allès B., Méjean C., Deschasaux M., Fassier P., Latino-Martel P., Beslay M. (2018). Consumption of ultra-processed foods and cancer risk: Results from NutriNet-Santé prospective cohort. BMJ.

[55] Gutiérrez S., Svahn S.L., Johansson M.E. (2019). Effects of omega-3 fatty acids on immune cells. Int. J. Mol. Sci..

[56] Srour B., Fezeu L.K., Kesse-Guyot E., Allès B., Debras C., Druesne-Pecollo N., Chazelas E., Deschasaux M., Hercberg S., Galan P. (2020). Ultraprocessed food consumption and risk of type 2 diabetes among participants of the NutriNet-Santé prospective cohort. JAMA Intern. Med..

[57] Oppedisano F., Macrì R., Gliozzi M., Musolino V., Carresi C., Maiuolo J., Bosco F., Nucera S., Zito M.C., Guarnieri L. (2020). The anti-inflammatory and antioxidant properties of n-3 PUFAs: Their role in cardiovascular protection. Biomedicines.

[58] Virsangbhai C.K., Goyal A., Tanwar B., Sihag M.K. (2020). Potential health benefits of conjugated linoleic acid: An important functional dairy ingredient. Eur. J. Nutr. Food Saf..

[59] Yang B., Chen H., Stanton C., Ross R.P., Zhang H., Chen Y.Q., Chen W. (2015). Review of the roles of conjugated linoleic acid in health and disease. J. Funct. Foods.

[60] Du M., Jin J., Wu G., Jin Q., Wang X. (2023). Metabolic, structure-activity characteristics of conjugated linolenic acids and their mediated health benefits. Crit. Rev. Food Sci. Nutr..

[61] Pierre A.-S., Minville-Walz M., Fèvre C., Hichami A., Gresti J., Pichon L., Bellenger S., Bellenger J., Ghiringhelli F., Narce M. (2013). Trans-10, cis-12 conjugated linoleic acid induced cell death in human colon cancer cells through reactive oxygen species-mediated ER stress. Biochim. Biophys. Acta Mol. Cell Biol. Lipids.

[62] Heinze V.M., Actis A.B. (2012). Dietary conjugated linoleic acid and long-chain n-3 fatty acids in mammary and prostate cancer protection: A review. Int. J. Food Sci. Nutr..

[63] Tanmahasamut P., Liu J., Hendry L.B., Sidell N. (2004). Conjugated linoleic acid blocks estrogen signaling in human breast cancer cells. J. Nutr..

[64] Kozloski G.V. (2017). Bioquímica dos Ruminantes.

[65] Dalli J., Zhu M., Vlasenko N.A., Deng B., Haeggström J.Z., Petasis N.A., Serhan C.N. (2013). The novel 13S, 14S-epoxy-maresin is converted by human macrophages to maresin 1 (MaR1), inhibits leukotriene A4 hydrolase (LTA4H), and shifts macrophage phenotype. FASEB J..

[66] Khadge S., Thiele G.M., Sharp J.G., McGuire T.R., Klassen L.W., Black P.N., DiRusso C.C., Cook L., Talmadge J.E. (2018). Long-chain omega-3 polyunsaturated fatty acids decrease mammary tumor growth, multiorgan metastasis and enhance survival. Clin. Exp. Metastasis.

[67] Dydjow-Bendek D., Zagoźdźon P. (2020). Total dietary fats, fatty acids, and omega-3/omega-6 ratio as risk factors of breast cancer in the polish population—A case-control study. In Vivo.

[68] Hanson S., Thorpe G., Winstanley L., Abdelhamid A.S., Hooper L. (2020). Omega-3, omega-6 and total dietary polyunsaturated fat on cancer incidence: Systematic review and meta-analysis of randomised trials. Br. J. Cancer.

[69] Nindrea R.D., Aryandono T., Lazuardi L., Dwiprahasto I. (2019). Protective effect of omega-3 fatty acids in fish consumption against breast cancer in Asian patients: A meta-analysis. Asian Pac. J. Cancer Prev..

[70] Ponnampalam E.N., Butler K.L., Pearce K.M., Mortimer S.I., Pethick D.W., Ball A.J., Hopkins D.L. (2014). Sources of variation of health claimable long chain omega-3 fatty acids in meat from Australian lamb slaughtered at similar weights. Meat Sci..

[71] Alfaia C., Ribeiro V., Lourenço M., Quaresma M., Martins S., Portugal A., Fontes C., Bessa R., Castro M., Prates J. (2006). Fatty acid composition, conjugated linoleic acid isomers and cholesterol in beef from crossbred bullocks intensively produced and from Alentejana purebred bullocks reared according to Carnalentejana-PDO specifications. Meat Sci..

[72] Oliveira A.C., Silva R.R., Oliveira H.C., Almeida V.V., Garcia F., Oliveira U.L.C. (2013). Influence of diet, sex and genotype on the lipid profile of sheep meat. Arch. De Zootec..

[73] Azmi A.F.M., Amin F.M., Ahmad H., Nor N.M., Meng G.Y., Saad M.Z., Abu Bakar Z., Abdullah P., Irawan A., Jayanegara A. (2021). Effects of bypass fat on buffalo carcass characteristics, meat nutrient contents and profitability. Animals.

[74] Renna M., Brugiapaglia A., Zanardi E., Destefanis G., Prandini A., Moschini M., Sigolo S., Lussiana C. (2019). Fatty acid profile, meat quality and flavour acceptability of beef from double-muscled Piemontese young bulls fed ground flaxseed. Ital. J. Anim. Sci..

[75] Costa C., Rizzieri R., Melo G., Müller L., Estevan D., Pacheco R., Millen D., Pereira A., Zanatta M., Cappellozza B. (2020). Effects of fatty acid profile of supplements on intake, performance, carcass traits, meat characteristics, and meat sensorial analysis of feedlot Bos indicus bulls offered a high-concentrate diet. Transl. Anim. Sci..

[76] Cappello A.R., Dolce V., Iacopetta D., Martello M., Fiorillo M., Curcio R., Muto L., Dhanyalayam D. (2016). Bergamot (*Citrus bergamia* Risso) flavonoids and their potential benefits in human hyperlipidemia and atherosclerosis: An overview. Mini Rev. Med. Chem..

[77] Zárate A., Manuel-Apolinar L., Saucedo R., Hernández-Valencia M., Basurto L. (2016). Hypercholesterolemia as a risk factor for cardiovascular disease: Current controversial therapeutic management. Arq. Pesqui. Méd..

[78] Diler A., Yanar M., Özdemir V.F., Aydin R., Kaynar Ö., Palangi V., Lackner M., Koçyigit R. (2022). Effects of Slaughter Age of Holstein Friesian Bulls on Meat Quality: Chemical Composition, Textural Characteristics, Sensory Attributes and Fatty Acid Profile. Foods.

[79] Orkusz A. (2021). Edible insects versus meat—Nutritional comparison: Knowledge of their composition is the key to good health. Nutrients.

[80] Simonetto M., Infante M., Sacco R.L., Rundek T., Della-Morte D. (2019). A novel anti-inflammatory role of omega-3 PUFAs in prevention and treatment of atherosclerosis and vascular cognitive impairment and dementia. Nutrients.

[81] Turan H., Sönmez G., Kaya Y. (2007). Fatty acid profile and proximate composition of the thornback ray (*Raja clavata*, L. 1758) from the Sinop coast in the Black Sea. J. Fish. Sci..

[82] Bentes A.S., de Souza H.A.L., Simões M.G., Mendonça X.M.F.D. (2009). Caracterização física e química e perfil lipídico de três espécies de peixes amazônicos. Rev. Bras. De Tecnol. Agroindustrial.

[83] Joo S.T., Hwang Y.H., Frank D. (2017). Characteristics of Hanwoo cattle and health implications of consuming highly marbled Hanwoo beef. Meat Sci..

[84] Bobe G., Zimmerman S., Hammond E.G., Freeman A.G., Lindberg G.L., Beitz D.C. (2004). Texture of butters made from milks differing in indices of atherogenicity. Iowa State Univ. Anim. Ind. Rep..

[85] Djuricic I., Calder P.C. (2021). Beneficial outcomes of omega-6 and omega-3 polyunsaturated fatty acids on human health: An update for 2021. Nutrients.

[86] Enser M., Halletta K.G., Hewetta B., Furseya G.A.J., Wooda J.D., Harrington A.N., Silva I.M.C. (2019). Imunoterapia: Um Olhar na nova modalidade terapêutica do Câncer. Rev. Multidiscip. Psicol..

[87] Bomfim J.H.G.G., da Silveira Gonçalves J. (2020). Suplementos alimentares, imunidade e COVID-19: Qual a evidência?. Vittalle-Rev. Ciências Saúde.

[88] Von Schacky C. (2020). Omega-3 fatty acids in pregnancy—The case for a target omega-3 index. Nutrients.

[89] Golanski J., Szymanska P., Rozalski M. (2021). Effects of omega-3 polyunsaturated fatty acids and their metabolites on haemostasis-current perspectives in cardiovascular disease. Int. J. Mol. Sci..

